# Pulmonologist-Administered Balanced Propofol Analgosedation during Interventional Procedures: An Italian Real-Life Study on Comfort and Safety

**DOI:** 10.1155/2022/3368077

**Published:** 2022-06-13

**Authors:** Rosalba Maffucci, Uberto Maccari, Luca Guidelli, Lucia Benedetti, Roberto Fabbroni, Bruno Piccoli, Andrea Bianco, Raffaele Scala

**Affiliations:** ^1^Division of Respiratory Physiopathology and Rehabilitation, A.O.R.N. “Dei Colli”—Monaldi Hospital, Naples, Italy; ^2^Pulmonology and Respiratory Intensive Care Unit, S Donato Hospital Arezzo, Cardio-Neuro-Thoracic and Metabolic Department, Usl Toscana Sudest, Arezzo, Italy; ^3^Consumer & Sensory Scientist, Adacta International, Naples, Italy; ^4^Department of Translational Medical Sciences, University of Campania “L. Vanvitelli”, Naples, Italy

## Abstract

Propofol-based sedation provides faster recovery than midazolam-based regimens with similar safety and comfort during video flexible bronchoscope (VFB) procedures. Pulmonologist-administered propofol “balanced” analgosedation (PAP-BAS) is still debated in Italy. In this real-life study, PAP-BAS safety and comfort during VFB procedures were investigated. We analysed prospectively the subjects undergoing elective VFB procedures in the Pulmonology and RICU of Arezzo Hospital between February and July 2019. PAP-BAS combined low propofol and meperidine doses titrated to achieve an RASS score between 0 and −3. The primary end-point was the complications' rate. Secondary end-points were as follows: the relation between propofol's dose and a subject's comfort assessed with a VAS, recovery time according to a modified Aldrete score ≥9, RASS, and subjects' will of undergoing the procedure again. We collected postprocedure symptoms' intensity too. Our 158 study patients (67 years; SD ± 14; 64% males) incurred in 25% of complication, fully resolved with medical therapy. Neither recourse to ventilator support nor death was reported. Intraprocedural comfort was good (94% of VAS score ≤2). Among postprocedural symptoms, cough was the most frequently reported, in 36% of the cases. Although half of subjects remembered the procedure, 90% of them would have repeated it, if necessary. 85% of them recovered from procedures within 10 minutes. Complications, VAS, and recovery time were not correlated with propofol dose. To our knowledge, this is the first Italian study showing that PAP-BAS to perform a VFB procedure is safe, well tolerated with a quick recovery. Randomised controlled trials are warranted to confirm these preliminary results.

## 1. Introduction

Sedation is recommended for subjects undergoing bronchoscopic procedures (BP) unless contraindications exist [1]. In the literature, there is no agreement about the best pharmacological strategy in sedation protocols [2, 3].

Propofol-based sedation is associated with reduced recovery time and not significantly different subjects' comfort and safety profiles, compared to midazolam-based regimens [4, 5].

Using propofol or midazolam is not strictly related to the level of the planned sedation but on the skills and expertise of the physician and on the impact on the subject's respiratory drive and hemodynamics during BP.

In several countries, it is not unanimous whether analgosedation should be performed only by anesthesiologist to grant safety [6]. According to the National Agency of Drugs (AIFA) in Italy, the use of propofol is allowed not only by anaesthesiologists but also by other physicians with large expertise in ICU [7]. Due to the risk of airways collapse, respiratory drive depression and negative hemodynamic effects, nonanaesthesiologists who decide to use this drug should have acquired skills in advanced airway management, mechanical ventilation, and treatment of cardiopulmonary complications. As a matter of the fact, administration of propofol by nonanaesthesiologists could not be considered a law violation in terms of specialty competence and subjects rights to safety. In Italy, training in ICU has been inserted in the time course of the specialty in pulmonology [8]. Moreover, pulmonologists working in RICUs who are mainly included in respiratory units are expert in the management of the pulmonary critically ill subjects [9]. Only few published data reported the safe and feasible use of propofol sedation during BP run by a team of pulmonologists and nurses adequately trained [10–16].

A non-ICU sedationist should have appropriate training in drug titration and resuscitation, should be dedicated to the task, and should avoid anesthesia and concomitant opioid administration. In Italy, there is no need of achieving a formal certification for nonanaesthesiologists to be allowed to perform procedural sedation.

The team of Pulmonology and RICU of S. Donato Hospital in Arezzo has gained a long experience with propofol and meperidine analgosedative protocol after attending specific courses tailored to develop practical and theoretical knowledge in using drugs for sedation; moreover, the capability of managing potential cardio-pulmonary complications of analgosedation was obtained after acquiring skills in the management of airways, mechanical ventilation, and BP in critically ill subjects. ICU physicians would have been always available if necessary for complicated patients. Since 2014, a pharmacological regimen including propofol in place of midazolam associated with meperidine has been introduced to perform all elective BP in the BU to reduce the recovery time and improve the subject's comfort.

Therefore, we performed a prospective “real-life” study assessing safety and satisfaction of conscious PAP-BAS based on the use of propofol and meperidine during BP, under the exclusive management of an expert pulmonologist team.

## 2. Material Methods

### 2.1. Setting and Selection Criteria

This is a prospective 6-month (February–July 2019), monocentric, nonprofit study including consecutive adult subjects, outward and inward, undergoing elective BP performed with video-fiber-bronchoscope (VFB) for diagnostic and therapeutic purposes (BAL, BLB/TBLB, TBNA, EBUS/TBNA, and endobronchial laser-therapy), at the BU of the Pulmonology and RICU, S. Donato Hospital, Arezzo, Italy.

All procedures were performed by two pulmonologists with the support of two nurses.

Exclusion criteria were

1. Hypersensitivity to propofol and meperidine
2. Requirement of noninvasive respiratory support (HFNC or NIV) or invasive mechanical ventilation (PaO_2_/FiO_2_ < 300 and/or pH < 7.35 with PaCO_2_ > 45 mm Hg while in oxygen therapy) [16]
3. Inability to spontaneously protect airways and managing bronchial secretions
4. Haemodynamic cardiovascular instability (i.e., requiring vasoactive amines and/or inotropic drugs)
5. Life-threatening arrhythmias requiring treatment
6. Acute coronary syndrome within 4–6 weeks
7. Psychomotor agitation requiring sedation before interventional procedures (i.e., RASS > 0)
8. Emergent interventional procedures
9. Pregnancy
10. Inability to obtain the subject's informed consent or refusal to take part in the study

### 2.2. Study Protocol

All subjects were monitored with noninvasive blood pressure measurements, ECG, SpO_2_, and respiratory and pulse rate. A venous access was positioned for administration of analgesics/sedatives and other drugs. Supplementary oxygen was provided by a Venturi mask titrated to guarantee SpO2 94–98% in hypoxemic and 88–92% in hypercapnic subjects.

All subjects received local anesthesia with 2% lidocaine, applied to the oropharynx and nasal mucosa with dropper instillation, and “spray-as-you-go technique” (during procedures) at a maximum of 7 mg/kg/body weight. Either nasal or oral access was used for performing a VFB procedure.

The PAP-BAS consisted of an initial bolus of 0.5 mg/kg/body weight of both propofol 1% (Diprivan Astra-Zeneca, Stockholm, Sweden) and meperidine, followed by a drip of propofol at the rate of 0.5–1.5 mg/kg/body weight/h.

Step-up and step-down propofol rate infusion was carried out according to changes in values of RASS considering a target range varying from −1 and −3, keeping subject's airways patent and effective spontaneous ventilation.

Insufficient sedation (e.g., pain or discomfort and agitation) required additional bolus of propofol and eventually meperidine (10–20 mg, till an overall maximum dose of 1 mg/kg/body weight).

The BU team (>10-year experience in VFB procedures, airway management, and critical care emergencies), consisted in a pulmonologist and two expert nurses, monitoring the subject's vital parameters, providing sedation and other drugs and assisting the pulmonologist in BP.

Recorded complications were defined as adverse events (AEs) and severe AEs (SAEs).

AEs included the following events:

- Hypotension: SBP < 90 mm Hg or DPB < 50 mm Hg in three consecutive measurements, requiring volume filling and/or vasoactive amines
- Hypertension: SBP > 170 mm Hg or DBP > 100 mm Hg in three consecutive measurements requiring hypotensive therapy
- Hypoxemia: SpO_2_ < 90% for more than 1 minute despite optimised oxygen support
- Arrhythmia: requiring treatment
- Bronchospasm: wheezing resolving with bronchodilator and/or systemic steroid therapy

SAEs included the following events:

- Death within 24 h after bronchoscopy
- Pneumothorax
- Major bleeding (necessity for intubation or placement of a bronchus blocker) and need of NIV or IMV
- Epileptic seizure
- Any event leading to an ICU admission after the procedure

In case of severe complications during/after BP, the oncall anesthesiologist-intensivist of the hospital was quickly available.

**Figure 1:**
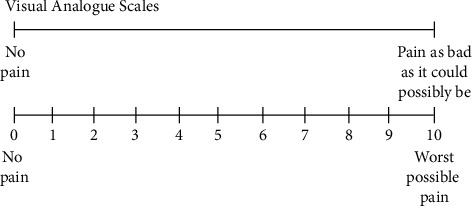
Visual Analog Scale (VAS).

When discharged from the BU, the subject's satisfaction was assessed with a Visual Analog Scale (VAS: from 1 for the best to 10 for the worst comfort) (Figure 1), [17] and an anonymous Likert's scale-type questionnaire about the persistence of symptoms reported post-BP (Table 1).

Recovery time from analgosedation and a safe discharge from the BU was defined by the time to reach the target of the modified Aldrete score ≥9. The Aldrete scoring system (minimum 0, maximum 10) is the most widely used tool to clinically assess subjects' physical status after anesthesia [18]. It evaluates the subject's consciousness, activity, respiration, blood pressure, and SpO_2_ (Table 2), [19]. A score between 0 and 2 is given for each of these five items.

The study was conducted in accordance with ethical principles and good clinical practice.

The study protocol received the approval of the local ethics committee.

### 2.3. End Points and Collected Data

The study's primary end-point was the periprocedural complications' rate.

Secondary endpoints were relation between a dose of propofol and VAS subject's comfort, the modified Aldrete score, RASS, route of bronchoscope access, and will of undergoing the procedure again, if necessary.

We also collected postprocedure symptoms evaluated with an anonymous Likert's scale-type questionnaire and all subjects' clinical conditions and BP's data.

### 2.4. Statistical Analysis

Descriptive statistics were indicated as frequency, mean value, and standard deviation (SD). A *p* value of <0.05 was considered significant for statistical evaluation.

The ordinal association among the propofol total dose and VAS, RASS, minute of the modified Aldrete score, and subject's will of undergoing the procedure again was measured with Kendall rank correlation coefficient (*τ*).

Pearson chi-squared test (*χ*^2^) was used to verify relationship among the propofol total dose and complications' rate and oral or nasal access during the VFB procedure.

Statistical analyses were performed using Statistical Package for the Social Sciences (SPSS, IBM, 26.0).

## 3. Results

During the study time, 162 consecutive subjects were analysed excluding four due to ventilator support before BP and missing data.

Demographic data, pre-VFB, and post-VFB physiological parameters were collected from the remaining 158 subjects (Table 3).

AEs occurred in 25% of the cases: hypoxemia (30%), hypotension (24%), and bronchospasm (22%). No SAEs were reported. All complications fully resolved after the administration of therapy in the BU (Table 4). There was no need to call the physician of the anesthesiology-ICU team for uncontrollable situation.

Subject satisfaction was good with 94% of cases showing VAS ≤ 2.

**Figure 2:**
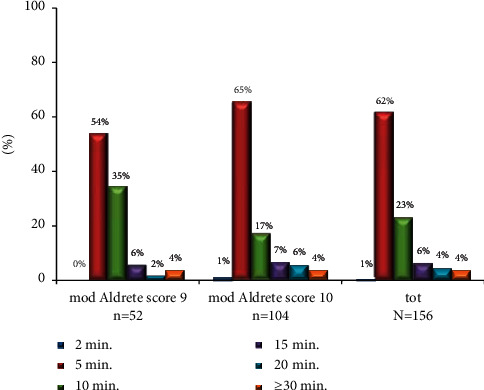
Time course of mod. Aldrete score: all subjects reached the modified Aldrete score ≥9 after VFB procedures.

All subjects reached the modified Aldrete score ≥9, with a recovery time of 5 and 10 minutes in 62% and 23% of subjects, respectively (Figure 2).

The mean administered doses of propofol and meperidine were, respectively, 57 mg (SD ± 26) and 57 mg (SD ± 25). The median speed of propofol infusion was of 5 mg/kg/body weight/min (SD ± 2).

AEs, VAS score, recovery time, route of bronchoscope access, and subjects' will of undergoing the procedure again were not correlated with the propofol dose. The study showed an inverse significant correlation between the propofol total dose and RASS, the higher was the propofol dose the deeper was the sedation (Table 5).

The mean RASS score was of −2 (SD ± 1); in 92%, a moderate/conscious sedation was reached.

68% of subjects had postprocedure persistent symptoms. Cough was reported in 36% of cases, with mild to moderate degree in 87% of cases. 50% had procedure memories; however, 90% of them would undergo the exam again, if required.

## 4. Discussion

To the best of our knowledge, our study is the first performed study in Italy and designed to assess prospectively the feasibility and safety of PAP-BAS for BP in spontaneously breathing subjects administered by pulmonologists during BP.

BP are commonly performed by pulmonologists as the gold standard technique for directly visualizing the airways; this procedure is performed under sedation in absence of contraindication [20]. Midazolam and opioids are currently used for BP, with the inclusion of EBUS-TBNA due to their easy application and rapid onset with good subject's satisfaction [21, 22]. However, the midazolam effect may have a delayed recovery time [23]. Conversely, propofol-based sedation has demonstrated good tolerance and quick recovery time after BP. This explains why propofol has been increasingly used to provide deep sedation in the BU showing similar effects in sedation, amnesia, and subject satisfaction, when compared with benzodiazepines [6].

However, due to its narrow therapeutic window and the lack of an antagonist drug, propofol is advised to be managed only by anaesthesiologists or physicians with great expertise in ICU and anesthesiological issues [24].

Only few published data dealt with NAAP protocol by pulmonology during BP. Moreover, most of the studies were based on the administration of high doses of propofol alone with an increased risk of oversedation, airway collapse, and severe respiratory failure requiring ventilator support. As a consequence, lower doses of propofol have been introduced in sedation protocols combining propofol with midazolam or meperidine (ie: *P-BAS*) which may be safely managed by nonanesthesiologists adequately trained in the management of airways collapse and cardiopulmonary complications [25]. pulmonologists working in RICU must be expert in mechanical ventilation, advanced airways management, sedation, as well as BP [9]. Previous experience demonstrated that at least some pulmonologists-running RICUs in Italy have become autonomous in performing endotracheal intubation [26].

Our study demonstrated the feasibility and safety of a PAP-BAS protocol performed by a team of pulmonologists and nurses with experience in managing different challenging situations in RICUs [27]. Complications were reported in 25% of the cases and were fully reversible without recourse to mechanical ventilation.

To our best knowledge, potential reversible cardiopulmonary changes occur in more than one third of patients submitted to bronchoscopic procedures due to several mechanisms (reduction of tracheal lumen, sympathetic system stimulation, alveolar filling, suction, and bleeding) [28]. It may be speculate that a balanced analgosedation is likely to be effective in preventing at least some distressed-induced complications during bronchoscopy.

These data agree with the findings of previous studies assessing the effects of AAPS alone or compared to midazolam protocols [10–12, 15, 29]. Mercado et al. experimented PAP-BAS in BP. In this randomised controlled trial, the authors compared NAAP (propofol and nalbuphine sedation) with midazolam and nalbuphine protocol, in terms of ventilatory response measured by transcutaneous capnometry (TcPCO2) in adult subjects undergoing ambulatory VFB procedure. NAAP-balanced sedation was not associated with increased TcPCO_2_ levels or a significant incidence of periprocedural AE, when administered by pulmonologists and critical care residents [13]. In a retrospective study, Muller et al. reported their encouraging experience with sedative regimens including a combination of propofol, midazolam, and fentanyl managed by pulmonologists with experience in critical care emergencies [15].

In our study, the average dose of propofol and meperidine administrated was 57 mg (SD ± 26) and 57 mg (SD ± 25), respectively, considerably lower than that used in protocols without concomitant opioid-based analgesia [12, 14] and also lower than studies using fentanyl or other opioid alone [10, 13]. These findings were in line with published data where midazolam and propofol doses were lower in studies combining sedatives with opioids in BAS [13, 30, 31], as compared to protocols based on “pure sedation” without concomitant analgesia [10, 14, 29, 32]. Muller et al. compared double sedation with either midazolam/fentanyl or midazolam/propofol to triple sedation with midazolam/fentanyl/propofol. Thereby they were able to show that subjects belonging to the triple sedation group received lower midazolam and propofol doses compared to the others [15].

Sedative protocols in BP are reported to improve the subject's comfort and tolerance during procedures. The majority of data on analgosedative usage were obtained by retrospective studies, with limited subject's comfort information [5, 15, 16, 33]. Agostini et al. investigated the subject's satisfaction in a sedative protocol using midazolam and meperidine in EBUS/TBNA procedures. In their work, they noticed a very high subject comfort [3].

Our prospective data agree with the published data about tolerance, comfort, and inclination of repeating the procedures, if necessary.

The economic impact of VFB sedation deserves a cost-utility analysis: even if the cost of propofol sedation may be higher than other protocols [14], it is reasonable to hypothesize that the PAP-BAS strategy without the anesthesiological assistance would have been cost-saving.

Our study has several limitations that deserve to be highlighted. Firstly, the study lacks control groups including subjects sedated with midazolam-based protocols by the same pulmonologist team. Secondly, safety and comfort of PAP-BAS required to be compared with the same propofol-based sedation performed by AAPS. However, our safety data are similar to the published ones on series where both strategies have been evaluated. Thirdly, the type and complexity of the procedures included in the study are heterogeneous in terms of depth of analgosedation required (i.e., EBUS-TBNA vs. BAL) that may have introduced a bias in the statistical analysis. Fourth, propofol regimen titration according to clinical end-points using a PK target-controlled infusion technique would have probably made the sedation easier and more accurate as well as safer as compared to the fixed dose of propofol (0.5 mg/kg) protocol followed in our study; this concept is based on the interindividual variability in pharmacodynamic response.

Lastly, the study was performed in a single center with highly experienced operators in performing PAP-BAS; so, these findings may be not reproduced in other centers with different training and skills in this issue.

The strength of our work is that it is the first real-life even if not controlled study in Italy specifically designed to prospectively assess the effects of a BAS protocol combining propofol and meperidine administered during BP and conducted by a trained team by pulmonologists, without continuous anesthesiologist assistance. This strategy, if confirmed by the controlled study, may have a relevant cost-saving impact in terms of anesthesiologist demand for what BP concerns.

## 5. Conclusions

Lack of specific guidelines for the appropriate level of sedation or personnel required to provide it for BP makes the role of the pulmonologist in managing sedation for advanced bronchoscopy unclearly defined.

The results of our study suggest that NAAP P-BAS, performed by an expert pulmonologist team, is not associated with a significant incidence of severe periprocedural AEs.

As the first prospective Italian “real-life” study, it evaluated safety and comfort for subjects undergoing these procedures, conjecturing that the association of propofol and meperidine can be a valid alternative to in use protocols.

Randomised controlled trials are required to assess the efficacy and safety of this pulmonologist-based analgosedation strategy as compared to anaesthesiologist-based sedation.

## Figures and Tables

**Table 1:** Anonymous Likert's scale-type questionnaire.

(1) Which of the symptoms listed below are you experiencing right now?	Cough	Sore throat	Chest pain	Shortness of breath	None	Others
(2) What is the intensity of the above symptom?	Very mild	Mild	Moderate	Moderate-intense	Intense	
	Totally agree	Agree	Uncertain	Disagree	In complete disagreement	
(3) The exam you have just undergone is as expected.						
(4) If necessary, you would repeat the procedure in the future.						
(5) You have (defined) memories of the procedure.						

**Table 2:** Modified Aldrete score.

Activity	0 min	5 min	15 min	20 min	30 min	40 min	50 min	60 min
Able to move 4 extremities voluntarily or on command (2 Points)								
Able to move 2 extremities voluntarily or on command (1 Point)								
Unable to move extremities voluntarily or on command (0 Points)								
Respiration								
Able to breathe deeply and cough freely (2 Points)								
Dyspnoea or limited breathing (1 Point)								
Apnoeic or need of mechanical ventilation (0 points)								
Circulation								
BP ± 20% of preanaesthetic level (2 Points)								
BP ± 20–49% of preanaesthetic level (1 Point)								
BP ± 50% of preanaesthetic level (0 Points)								
Consciousness								
Fully awake (2 Points)								
Arousable on calling (1 Point)								
Not responding (0 Points)								
O_2_ Saturation								
Able to maintain SpO_2_ >92% on room Air (2 points)								
Needs supplementary O_2_ to maintain SpO_2_ >90% (1 point)								
SpO_2_ <90% despite supplementary O_2_ (0 points)								

Patients who score 9 or greater and have an appropriate escort can go home.

**Table 3:** Demographic data and comorbidities and physiological parameters before and after VFB procedure.

Demographic data and comorbidities	*Mean (SD)*	
Age	67 (SD ± 14)	
Weight	71 (SD ± 14)	
	% (*n*)	
Male (%)	64 (101)	
Chronic respiratory failure	6 (15)	
COPD	9 (20)	
CAD	4 (10)	
CHD	2 (5)	
Systemic arterial hypertension	23 (54)	
Arrhythmias	6 (15)	
Diabetes mellitus	8 (19)	
Chronic liver disease	2 (59)	
Malignancies	11 (26)	
Chronic renal diseases (%)	9 (4)	

*Physiological parameters*	*Before VFB mean (SD)*	*After VFB mean (SD)*
SBP (mmHg)	137 (±23)	127 (±21)
DBP (mmHg)	75 (±13)	73 (±12)
HR (ppm)	78 (±14)	81 (±15)
SatO2 (%)	96 (±2)	95 (±3)
RR (bpm)	18 (±4)	17 (±4)

SD: standard deviation; age in years; weight in kg; COPD: chronic obstructive pulmonary disease; CAD: coronary artery disease; CHD: chronic heart disease; VFB: video-fiber-bronchoscope; SBP: systolic blood pressure; DBP: systolic blood pressure; HR: heart rate; SatO2: oxygen saturation; RR: respiratory rate.

**Table 4:** Incidence of AEs^*∗*^/SAEs^†^ and treatment of AEs.

Incidence of AEs/SAEs	% (*n*)
Hypoxemia	30 (15)
Hypotension	24 (12)
Bronchospasm	22 (11)
Hypertension	10 (5)
Minor bleeding	10 (5)
Arrhythmia	0 (0)
SAEs	0 (0)
Tot	100 (48)

*Treatment of AEs*	% (*n*)
Volume filling	22 (11)
Inhaled bronchodilatories	32 (16)
ICS	6 (3)
Systemic steroids	10 (5)
Diuretics	6 (3)
O_2_ extra supply	10 (5)
OT	8 (4)
Procoagulants	6 (3)

^*∗*^Adverse events/†severe adverse events. ICS: inhaled corticosteroids; OT: other therapies: adrenaline.

**Table 5:** Relation between propofol total dose and sedation depth, VAS, will of undergoing again to the procedure, minutes to reach the modified Aldrete score of ≥9, AEs.

Variable 1	Variable 2	Kendall correlation (*τ*)	CI (95%)		*p* value
Propofol total dose (mg)^‡^	RASS	−**0.250**	−0.348/−0.146		*0.001*
Propofol total dose (mg)^‡^	VAS	**0.108**	0.002/0.212		*0.144*
Propofol total dose (mg)^‡^	q.4^δ^	−**0.029**	−0.138/0.080		*0.704*
Propofol total dose (mg)^‡^	Min Aldrete	**0.001**	−0.106/0.105		*0.993*

		Pearson chi-squared (*χ*^2^)			
Variable 1	Variable 2	Coefficient		*p* value	
Propofol total dose (mg)^‡^	AEs	2.259		0.32	
Propofol total dose (mg)^‡^	VFB route	3.089		0.21	

RASS: Richmond Agitation Sedation Scale; VAS: Visual Analog Scale; ^δ^: Question 4 of anonymous questionnaire: subjects' will of undergoing the procedure again; min Aldrete: minutes to reach the modified Aldrete score of ≥9; AEs: adverse events/severe adverse events; VFB route: oral or nasal;^‡^ grouped in classes: 20–50 mg; 51–80 mg; >80 mg. Kendal coefficient expresses the correlation between total dose and every single data detected for VAS, RASS, minute of the modified Aldrete score, and subject's will of undergoing the procedure again. p value significance: 95%.

## Data Availability

All the findings of study, as well as the final data with tables and extra material, will be available for all the readers and researchers with interest in this issue.

## Abbreviations

AEs: Adverse events
BAL: Bronchioloalveolar lavage
BAS: Balanced analgosedation
BLB/TBLB: Bronchial/transbronchial biopsies
BP: Bronchoscopic procedures
BU: Bronchoscope unit
EBUS/TBNA: Endobronchial ultrasound/transbronchial needle aspiration
HFNC: High flow nasal cannula
ICU: Intensive care unit
NAAP: nonanaesthesiologist-administered propofol
NIV: Noninvasive ventilation
PaO_2_/FiO_2_: Pressure of arterial oxygen to fractional inspired oxygen concentration
PAP-BAS: Pulmonologist-administered propofol balanced analgosedation
P-BAS: Propofol balanced analgosedation
RASS: Richmond agitation sedation scale
RICU: Respiratory intensive care unit
SAEs: Severe adverse events
SD: Standard deviation
SpO_2_: Oxygen transcutaneous saturation
TBNA: Transbronchial needle aspiration
VAS: Visual analogic scale
VFB: Video flexible bronchoscope.
